# Supplementary material for: Identification of Freezing-Responsive microRNAs and Their Targets in Chinese Jujube by Small RNA and Degradome Sequencing
Source: Int J Mol Sci. 2026 Sep 4;27(17):7912. doi: 10.3390/ijms27177912 (PMC13566890; doi:10.3390/ijms27177912)
Supplement: Supplementary file 1 [file ijms-27-07912-s001.zip › Supplementary Material S2/novel_miR_199.pdf]

Provisional ID	:	novel_miR_199
Score total	:	0.6
Score for star read(s)	:	-1.3
Score for read counts	:	0
Score for mfe	:	0.3
Score for randfold	:	1.6
Score for cons. seed	:	
Total read count	:	337
Mature read count	:	337
Loop read count	:	0
Star read count	:	0

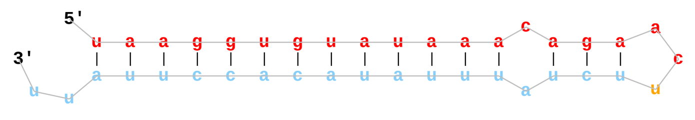

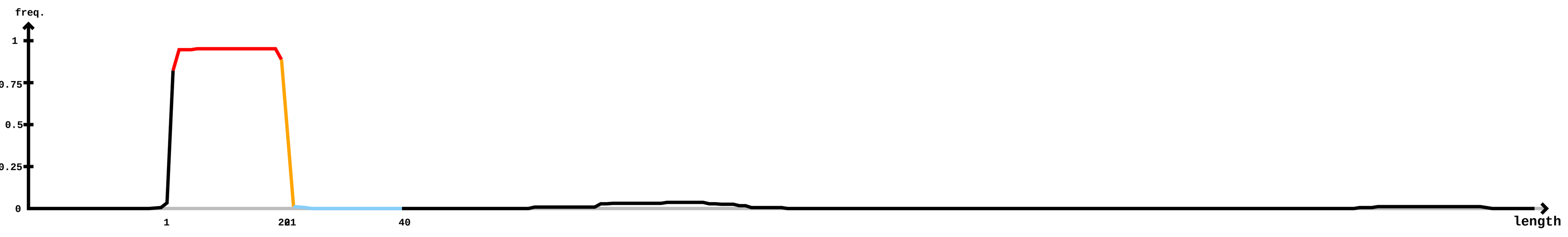

Mature Star

5'- gguagaagcaaacaaaaugaaauaagguguauaaacagaacuucuauuuauacaccuuauuuugcuuauucaaacccaccaccuaguuacgacaucacauugauagccucuacucguguccuacaaugguagggacaaagggucgcgaucccaugagagugagcuaacuugaaaaacucagacauauugaguacauccuauacacguaucauaaaucuuuauugaaugugacauuggaucuauugguucu -3' exp

...((((.(((.......((((((((((((((((.(((...))).))))))))))))))))))((((((((...((((((..(((.(((((...................)))))..)))...)))).))).....(((.......)))....))))))))..........((((((.....))))))..((((....(((((.(((...........))))))))).....))))...))).))))	reads	mm	sample
...................aaauaagguguauaaacagaac...	1	0	LT-1
....................aauaagguguauaaacagaa...	1	0	LT-3
.....................auaagguguauaaacagaac...	2	0	LT-1
.....................auaagguguauaaacagaac...	4	0	LT-2
.....................auaagguguauaaacagaac...	1	0	JCK-3
.....................auaagguguauaaacagaac...	2	0	LT-3
.....................auaagguguauaaacagaacuu...	1	0	JCK-2
......................uaagguguauaaacagaa...	1	0	JT-3
......................uaagguguauaaacagaa...	2	0	LT-2
......................uaagguguauaaacagaa...	7	0	LT-3
......................uaagguguauaaacagaa...	2	0	JCK-2
......................uaagguguauaaacagaa...	3	0	JT-2
......................uaagguguauaaacagaa...	1	0	JCK-1
......................uaagguguauaaacagaC...	5	1	LCK-3
......................uNagguguauaaacagaa...	1	1	JT-2
......................uaagguguauaaacagaac...	14	0	JT-1
......................uaagguguauaaacagaac...	26	0	LT-2
......................uaagguguauaaacagaac...	26	0	JCK-3
......................uaagguguauaaacagaac...	42	0	LT-1
......................Caagguguauaaacagaac...	1	1	JCK-1
......................uCagguguauaaacagaac...	1	1	LT-1
......................uaagguguauaaacagaac...	49	0	LCK-3
......................uaagguguauaaacagaac...	62	0	LT-3
......................uaagguguauaaacagaac...	17	0	JCK-1
......................uaagguguauaaacagaac...	17	0	JCK-2
......................uaagguguauaaacagaacA...	2	1	LT-1
......................uaagguguauaaacagaacu...	1	0	LT-1
.......................aagguguauaaacagaac...	2	0	JCK-3
.......................aagguguauaaacagaac...	6	0	LT-3
.......................aagguguauaaacagaac...	9	0	LT-2
.......................aagguguauaaacagaac...	10	0	LCK-3
.......................aagguguauaaacagaac...	8	0	LT-1
.......................aagguguauaaacagaacu...	2	0	LT-3
.......................aagguguauaaacagaacu...	2	0	LT-1

```
                Mature     Star
gguagaagcaaacaaaaugaaauaagguguauaaacagaacuucuauuuauacaccuuauuuugcuuauucaaacccaccaccuaguuacgacaucacauugauagccucuacucguguccuacaaugguagggacaaagggucgcgaucccaugagagugagcuaacuugaaaaacucagacauauugaguacauccuauacacguaucauaaaucuuuauugaaugugacauuggaucuauugguucu
.......................aagguguauaaacagaacA................................................................................................................................................................................................................   1   1   JCK-3
.......................aagguguauaaacagaacA................................................................................................................................................................................................................   2   1   JCK-2
.......................aagguguauaaacagaacA................................................................................................................................................................................................................   1   1   JCK-1
.......................aagguguauaaacagaCcuuc..............................................................................................................................................................................................................   1   1   JCK-2
..........................guguauaaacagaacuucu.............................................................................................................................................................................................................   2   0   LT-1
..................................................................................cuaguuacgaUaucacauugauagccucu...........................................................................................................................................   2   1   JCK-2
..................................................................................cuaguuacgaUaucacauugauagccucu...........................................................................................................................................   1   1   JCK-3
.............................................................................................aucacauugauagccucuacucg......................................................................................................................................   3   0   LCK-3
.............................................................................................aucacauugauagccucuacucguA....................................................................................................................................   4   1   JCK-2
...............................................................................................cacauugauagccucuac.........................................................................................................................................   1   0   LT-3
........................................................................................................agccucuacucguguccuCc..............................................................................................................................   2   1   LT-1
...........................................................................................................................................................................................................................uaCugaaugugacauuggauc..........   1   1   JCK-2
...........................................................................................................................................................................................................................uaCugaaugugacauuggauc..........   1   1   JCK-3
..............................................................................................................................................................................................................................ugaaugugacauuggaucu.........   2   0   LCK-3
```
